# Supplementary material for: Chromothripsis during telomere crisis is independent of NHEJ, and consistent with a replicative origin
Source: Genome Res. 2019 May;29(5):737–49. doi: 10.1101/gr.240705.118 (PMC6499312; doi:10.1101/gr.240705.118)
Supplement: Supplemental Material [file supp_gr.240705.118_Supplemental_file_1.zip › contigs/annotated_contigs/DB84/contig.2.DB84_length_634_mean_cov_7.57097791798.docx]

**DB84_length_634_mean_cov_7.57097791798**

```
CCA|TTTTTCAAACTCAAACTCTTCTAAATAGAAACAATGAAGATCCATTTAACTGCATTTATGTAAAATAAAATAAAACCCCAGATAC
    >chr4:126354202-126354574 - E=4e-212
TCTCCTTTTCTGCTTAAGATACTACTTTACCTTCAATAAAGTTATTTCCCTTAATATGACATTTAAATTCTACTTAACTTTACCATTCA

ATATTCTGTAAATAAAGCCACTCATTCATTTATTTGTAAAATGTTTATGGAGTGCCCTCATTATCACAGAATGGTATTGAGATTCACAA

CCTTACCATATTATTTTCTAATAGTCAAGTGATTTGCTGGTATTTCCTTTACCACTATTCAATCATTCTTCTATGTTCTTAGATTGTAA

TTACTCGTGGCCTAGTACCA|TAATGA|ACATTAATATTTGGTATTCTAAAGTGTCTGGTGATACACATATGGAAAACCACATTAAATT
                           >chr4:126352091-126352344 - E=2e-137
ATGACTGACTCATTTGGACAAGAATATGTGATTTCTTTCTTTGTCTGTAAACCATATATTACTGTAGATACATTACTACTGTAGATACA

TTACTGTAGATACTGTAGAAAGCAAATGCTGGGGGGAGTAAAAAATGAGCCCTGTTTATCAAGGAAAGCTTACAGCAAGTTCAGTCTCA

CTGTGTTCATACCA
```
